# Supercapacitor Electrodes: Is Nickel Foam the Right Substrate for Active Materials?

**DOI:** 10.3390/ma17061292

**Published:** 2024-03-11

**Authors:** Milena P. Dojčinović, Ivana Stojković Simatović, Maria Vesna Nikolić

**Affiliations:** 1 Institute for Multidisciplinary Research, University of Belgrade, Kneza Višeslava 1, 11030 Belgrade, Serbia; mariavesna@imsi.bg.ac.rs; 2 Faculty of Physical Chemistry, University of Belgrade, Studentski trg 12–16, 11000 Belgrade, Serbia; ivana@ffh.bg.ac.rs

**Keywords:** Ni foam, nickel oxide, nickel hydroxide, supercapacitors, substrate, alkaline media

## Abstract

Ni foam is an extensively used current collector and substrate in investigations of electrochemically active materials such as supercapacitors and electrocatalysts for oxygen and hydrogen evolution reactions. This material is relatively cheap, porous, and conductive and has a large specific surface area, all of which make it a good substrate. We investigated Ni-Mg ferrites and NiMn_2_O_4_ as active materials for electrochemical energy storage. These materials, when loaded on Ni foam, gave promising capacitance values: 172 F/g (at 2 mV/s) for NiMn_2_O_4_ in 6 M KOH and 242 F/g (at 2 mV/s) for MgFe_2_O_4_ in 3 M KOH. Nevertheless, during the authors’ work, many experimental problems occurred. Inconsistencies in the results directed further investigation towards measuring the capacitance of the active materials using GCE and platinum electrodes as substrates to discover if Ni foam was the culprit of the inconsistencies. When non-nickel substrates were used, both NiMn_2_O_4_ and MgFe_2_O_4_ showed reduced capacitance. Experimental problems associated with the utilization of Ni foam as a substrate for active materials in supercapacitor electrodes are discussed here, combined with other problems already addressed in the scientific literature.

## 1. Introduction

In recent decades, electronic equipment has mostly become portable. This has induced a need for electric energy storage devices that must meet specific requirements regarding output power, voltage, and current, safety, ability to recharge, etc. Utilization of abundant, non-toxic, cheap, and recyclable materials for the production of energy storage devices is encouraged. Investigations of the materials’ ability to store and release electric energy are mostly conducted in a similar way [1,2,3]. Research articles usually investigate energy storage properties via cyclic voltammetry and galvanostatic charge/discharge cycling in a three-electrode cell and include performance and stability tests of formed supercapacitor and battery devices. However, not all experimental configurations are identical, and the utilization of certain setups is often inadequately acknowledged and comprehended. Nickel electrodes are an essential part of both scientific and industrial applications. They are used in batteries [4,5,6,7,8], fuel cells [9,10,11], and as electrocatalysts in hydrogen and oxygen evolution reactions (HERs and OERs) [12,13,14,15,16]. Ni foam is a popular porous substrate praised for its conductivity, high specific surface area, and low cost. It is often used when investigating active materials for supercapacitor applications, mainly as a substrate for metal oxides as anodes [17,18,19,20,21,22,23,24,25,26,27,28,29,30,31,32,33,34,35,36,37,38,39,40,41]. In this work, Ni foam was used as a substrate for NiMn_2_O_4_ and Ni-Mg ferrites to investigate their energy storage properties. These materials were tested both on Ni foam and planar electrodes. We encountered and elaborated on several experimental problems that occurred with Ni foam usage. Firstly, Ni foam may undergo electrochemical redox changes along with the investigated active material. Redox reactions of nickel are well described in the literature [16,42,43,44,45,46,47]. Secondly, nearly all of the scientific articles that use Ni foam as a substrate claim that the cleaning of Ni foam before loading it with active material is necessary to remove surface oxides and expose the metallic nickel surface. Even so, we discovered that exposure to acid may cause the corrosion of nickel and increase surface roughness, which affects charge storage, and generates oxides that may be electroactive. Other problems with Ni foam are noted in the recent literature. Zheng et al. [48] expressed concern that electrocatalyst benchmarks lose their meaning when porous electrodes are used as substrates because the current density used to evaluate electrocatalyst’s performance cannot be calculated when the electrode surface is not exactly defined. Therefore, the electrode surface has to be calculated through the capacitance of the double layer or by some other methodology [16,49]. Other problems with using Ni foam include dissimilarities of Ni foam specifications, the adequate immersion of porous Ni foam into the electrolyte, and contamination of the active material when Ni foam is used as a part of hydrothermal synthesis [48].

NiMn_2_O_4_ is a material with great potential for application in supercapacitors [20,21,22,23,24,25]. Recent research has focused on composite and asymmetric electrode setups, where NiMn_2_O_4_ is combined with carbonaceous materials or other metal oxides [50,51,52,53]. Cubic spinel ferrites, such as Ni-Mg ferrites, have demonstrated energy storage capability, and ongoing research considers the effect of composition and synthesis parameters on their supercapacitive properties [41,54,55,56]. NiMn_2_O_4_ and mixed Ni-Mg ferrites (Ni_x_Mg_1−x_Fe_2_O_4_) synthesized by sol–gel auto-combustion processes were therefore tested as supercapacitor materials on Ni foam as a substrate and on other substrates such as Pt and GCE. Comparison of the obtained results showed that Ni foam greatly influences the results. The usage of Ni foam as a substrate for active materials in supercapacitor investigations is discussed further in this article, through experimental results and consideration of the literature.

## 2. Materials and Methods

### 2.1. Materials

Ni foam was purchased from Goodfellow Cambridge Limited (Huntingdon, UK) with the following characteristics: thickness of 1.6 mm, bulk density of 0.45 g cm^−1^, porosity of 95%, 20 pores/cm, with purity of 99.5%. The chemicals used were as follows: KOH (85%, Sigma Aldrich, Merck, Darmstadt, Germany), HCl (37%, Zorka, Šabac, Serbia), ethanol (Honeywell, puriss p.a., min. 99.8%, Seetze, Germany), deionized water, Nafion (D-521 dispersion, 5% *w*/*w* in water and 1-propanol, Alfa Aesar, Kandel, Germany), carbon black (Vulcan XR-72, 50 nm spherical particles, Cabot, Boston, MA, USA), previously synthesized and characterized NiMn_2_O_4_ [57], and mixed Mg-Ni ferrites [58]. NiMn_2_O_4_ was synthesized via a sol–gel auto-combustion method using glycine as the fuel and chelating agent and nitrate ions as the oxidizing agents. After heating on a hot plate at 300 °C, which caused gel formation, followed by its combustion, the obtained black combusted powder was subjected to calcination at 800 °C, resulting in pure phase cubic spinel NiMn_2_O_4_. Thorough structural and morphological characterization was conducted, and the results were published in our previous paper [57]. Spinel Ni-Mg ferrites Ni_x_Mg_1−x_Fe_2_O_4_ with x = 0, 0.1, 0.3, 0.5, 0.7, 0.9, and 1 were synthesized via a sol–gel auto-combustion method with citric acid as fuel and nitrate ions as oxidizers accompanied by subsequent calcination at 700 °C. Thorough structural and morphological characterization was conducted, and the results were published in our previous paper [58]. A spinel Fd$\bar{3}$m structure was obtained for all of the synthesized materials, with the presence of some hematite in all of the samples (0–7%). Spinel ferrites had crystallite sizes in the range of 30–39 nm.

### 2.2. Electrode Preparation

Ni foam was cut into 3 × 1 cm^2^ strips. The cleaning of Ni foam was conducted by ultrasonicating Ni foam in 1 M HCl solution for 10 min, followed by rinsing with ethanol and deionized water afterwards. Electrode ink for the Ni foam substrate was made by ultrasonically dispersing 20 mg of the active material, carbon black, and Nafion in a weight ratio of 85:10:5 in 500 μL of ethanol for 1 h. When Ni foam was coated with the electrode ink, it was left overnight at 80 °C. Electrode ink for the planar electrodes was made by ultrasonically dispersing 20 mg of the active material, carbon black, and Nafion in a ratio of 85:10:5 in 100 μL of ethanol for 1 h, and then it was loaded on a clean and polished GCE and Pt electrodes and dried with infrared light (Infrared heating lamp 250 W, Avide, Düren, Germany) for 5 min. The cleaning effect of hydrochloric acid on Ni foam was investigated by comparing 10 consequential CV cycles in 3 M KOH of (1) untreated Ni foam, (2) Ni foam cleaned by ultrasonicating for 10 min in 1 M HCl, and for 10 min in ethanol, and (3) Ni foam cleaned by ultrasonicating for 30 min in 1 M HCl and for 30 min in ethanol.

### 2.3. Methods

NiMn_2_O_4_ was tested as an active material for charge storage application on Ni foam as a substrate, in 6 M KOH, similar to the published literature [21,22,23], and also on a GCE as a substrate. Ni-Mg ferrites were tested on Ni foam as a substrate, in 3 M KOH, as described in the literature [59], and also on GCE and Pt substrates. The electrochemical behavior of untreated, bare Ni foam was tested in the same three-electrode setup in 3 M KOH solution. Cyclic voltammetry (CV) was used as the main electrochemical method. Electrochemical measurements were performed with Ivium One Galvanostat Potentiostat/ZRA (Ivium Technologies, Eindhoven, The Netherlands). The three-electrode cell setup was composed of Ag/AgCl (3 M KCl) as a reference electrode and a platinum wire as a counter electrode in a glass electrochemical cell. The working electrodes were Ni foam, with GCE and Pt as substrates, loaded with active materials or bare. Ni foams were clipped with alligator clips and connected to the measuring device. The intersection of Ni foam and the alligator clip was covered in Teflon tape. In all measurements, 1 cm^2^ of the Ni foam was immersed in the electrolyte. Important note: all of the results are reported against a Ag/AgCl (3 M KCl) reference electrode, unless stated otherwise.

## 3. Results

### 3.1. Electrooxidation of Nickel—Experimental Results and Literature Consideration

**Figure 1:**
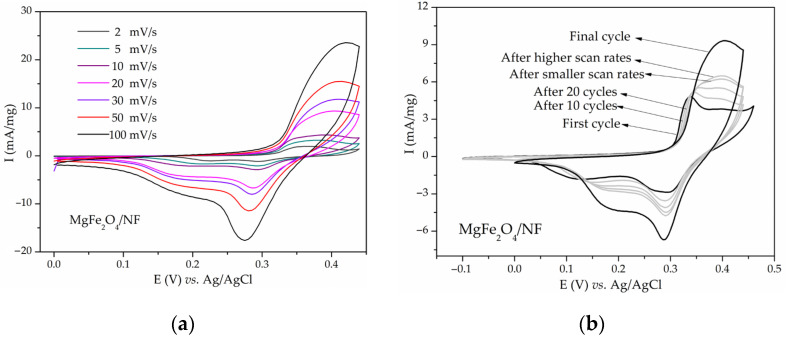
CVs of (**a**) MgFe_2_O_4_ on Ni foam at different scan rates; (**b**) MgFe_2_O_4_ on Ni foam after various numbers of cycles in 3 M KOH at 20 mV/s.

CVs of MgFe_2_O_4_ in 3 M KOH at different scan rates (2–100 mV/s) are shown in Figure 1a. CVs show a clearly differentiated anodic peak at 0.35–0.45 V and a cathodic peak at 0.25–0.28 V. The anodic peak position shifted to the right, while the cathodic peak position shifted to the left with an increase in the scan rate from 2 to 100 mV/s, as expected for a diffusion-controlled process [60]. Figure 1b shows the CV of MgFe_2_O_4_, while CVs of Ni_x_Mg_1−x_Fe_2_O_4_/NF with x being 0.1, 0.3, 0.5, 0.7, 0.9, and 1 recorded with a scanning rate of 20 mV/s are shown in Figure S1a–f, respectively. All of the CVs show redox peaks similar in shape and position to the peaks of MgFe_2_O_4_. The most notable feature is that the peaks in all of the CVs increased with the number of cycles. This is illustrated in Figure 1b and Figure S1 as a comparison between CVs of the first cycles and CVs after tens of stabilization cycles and final cycles. It is important to note that this increase did not disappear, even after 100 cycles. In some of the CVs, the cathodic peak split into two components with cycling. At first, the high current response was attributed to the electrochemical activity of Ni_x_Mg_1−x_Fe_2_O_4_. The highest obtained capacitance was 242 F/g for MgFe_2_O_4_/NF at a scanning rate of 2 mV/s. However, after learning that Ni foam may also participate in redox reactions in the same potential range [61,62], some of the samples were tested on platinum and GCE substrates.

**Figure 2:**
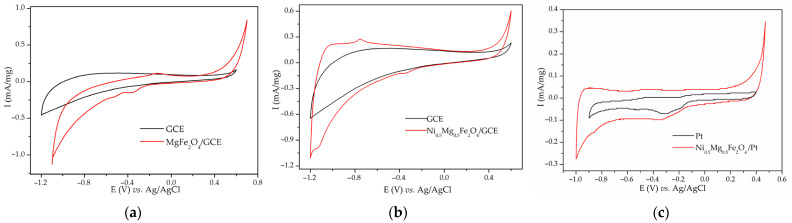
CVs of (**a**) MgFe_2_O_4_/GCE substrate, (**b**) Ni_0.5_Mg_0.5_Fe_2_O_4_/GCE, and (**c**) Ni_0.5_Mg_0.5_Fe_2_O_4_/Pt compared to the CVs of respective substrates in 3 M KOH at 20 mV/s.

CVs on a GCE were recorded in a potential range between −1.2 V and 0.5, 0.6, or 0.7 V, depending on the onset of HERs and OERs, with a scanning rate of 20 mV/s. Figure 2a,b show the CVs of MgFe_2_O_4_/GCE and Ni_0.5_Mg_0.5_Fe_2_O_4_/GCE compared to the CVs of bare GCE substrate. The calculated capacitance of MgFe_2_O_4_/GCE substrate was 8 F/g, while the calculated capacitance of Ni_0.5_Mg_0.5_Fe_2_O_4_/GCE was 7 F/g, which is multiple times lower than the values obtained when Ni foam was used (150 and 75 F/g at 20 mV/s). Both CVs show no peaks that were originally noticed when Ni foam was used as a substrate. Figure 2c shows a CV of Ni_0.5_Mg_0.5_Fe_2_O_4_/Pt compared to a CV of bare Pt. CV of the material, again, shows no peaks resembling the ones obtained with Ni_0.5_Mg_0.5_Fe_2_O_4_/NF (Figure S1c). Even after subduing active materials to more cycles, no new peaks appeared. Therefore, it was concluded that the investigated Ni-Mg ferrites are not electrochemically active in 3 M KOH, contrary to the original conclusions made when Ni foam was used as a substrate.

**Figure 3:**
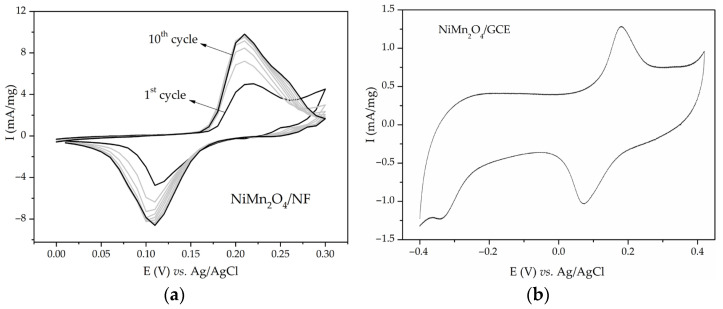
CVs of (**a**) NiMn_2_O_4_/NF and (**b**) NiMn_2_O_4_/GCE in 6 M KOH at 20 mV/s.

**Figure 4:**
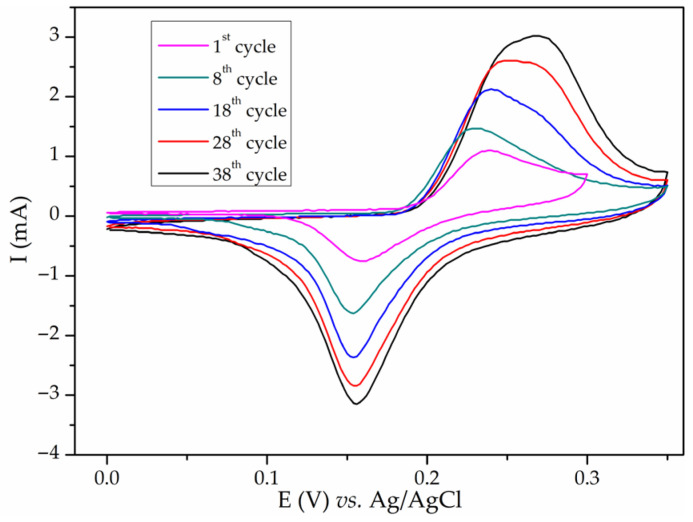
CVs of bare Ni foam cycled in 3 M KOH for various numbers of cycles at 20 mV/s.

The obtained CVs for NiMn_2_O_4_/NF in 6 M KOH after various numbers of cycles are shown in Figure 3a. CVs contain anodic and cathodic redox peaks, with the shapes and positions of the peaks being similar to the ones obtained for Ni_x_Mg_1−x_Fe_2_O_4_/NF. An increase in the redox peaks and cathodic peak split with the number of cycles was observed again. The measurement was repeated four times. The calculated capacitance values for four probes were 32, 64, 158, and 165 F/g at 20 mV/s. After obtaining inconsistent results, NiMn_2_O_4_ was tested on a GCE substrate with the same experimental setup. There was no current response in the CV of NiMn_2_O_4_/GCE in the first cycle, but after 10 cycles in the potential window −1–0.4 V, redox peaks emerged. After stabilization, this material was subdued to cycling in the potential window of −0.4–0.42 V (Figure 3b) since the current below −0.4 V was negligible. It is important to note that in this case, no further increase in peak intensity was noticed. The capacitance of NiMn_2_O_4_/GCE was calculated to be 33 F/g at 20 mV/s, which is much lower than NiMn_2_O_4_/NF (165 F/g at 20 mV/s). Since a modest capacitance value was obtained, it was concluded that NiMn_2_O_4_ synthesized via sol–gel combustion synthesis is electrochemically active. Its capacitance is due to electrochemical reactions of nickel, which will be discussed in more detail. Further research was directed to clarifying nickel foam behavior in alkaline media. Bare Ni foam, used “as received from the manufacturer”, was subjected to cycling in 3 M KOH in various potential intervals. Figure 4 shows various CVs (0.0–0.3 and 0.4 V). Redox peaks similar to the ones in Figure 1, Figure 2, and Figure S1 appeared. These peaks also grew with each cycle (Figure 4). This indicated that uncoated Ni foam was undergoing electrochemical redox processes in alkaline media. It is worth mentioning that before conducting the measurements on NiMn_2_O_4_, uncoated Ni foam underwent one voltammetric cycle in 6 M KOH. The resulting CV had no current response.

The obtained redox peaks in the CVs of NiMn_2_O_4_ and Ni_x_Mg_1−x_Fe_2_O_4_ were at first assigned to fast redox reactions of metal oxides [63]. The considerably high capacitance values are in line with the already published results [20,21,22,23,24,25,64]. While Ni_x_Mg_1−x_Fe_2_O_4_/NF have shown capacitance up to 242 F/g at 2 mV/s, they have shown no electrochemical activity when loaded on a GCE or Pt substrate (Figure 1, Figure 2, and Figure S1). It is worth mentioning that other researchers also obtained the capacitive performance of mixed Ni-Mg ferrites [41,64]. On the other hand, NiMn_2_O_4_/GCE exhibited some capacitance (33 F/g at 20 mV/s), but much less than the value obtained with NiMn_2_O_4_/NF (165 F/g at 20 mV/s) (Figure 3). Yadav et al. also obtained better capacitance results when Ni foam was used [65]. The conclusion can be reached that most of the current response actually originated from the redox reactions of Ni foam.

**Figure 5:**
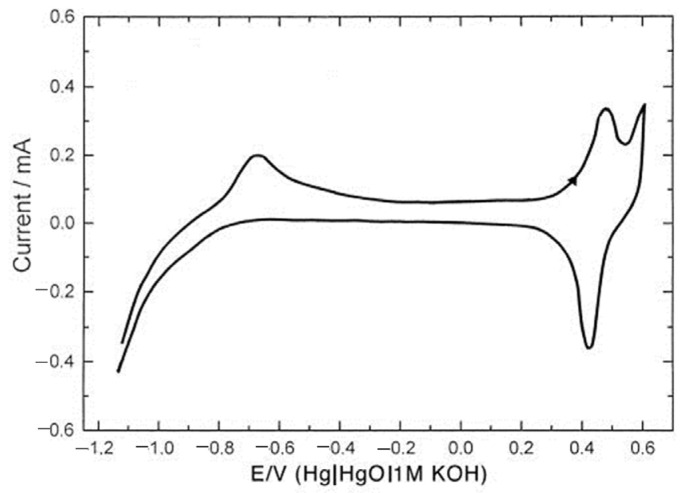
Voltammograms of Ni oxidation in 1 M KOH with a sweep rate of 50 mV/s. The arrow shows the direction of potential change. Reprinted with permission from Seghiouer et al. [56]. Publisher: Elsevier.

A literature review provides more insight into the electrochemistry of nickel in alkaline media. While nickel etches in acid solutions [42], it offers good corrosion resistance in alkaline media [16] because it quickly forms a passive oxide layer [44]. Nickel goes through oxidation $\mathrm{Ni}+2\;{\mathrm{OH}}^{-}⇌{\mathrm{Ni}(\mathrm{OH})}_{2}$ at the potentials around −0.865–−0.565 V, represented as the peak at the potential of −0.7 V vs. Hg/HgO in Figure 5 (note: figure republished with permission. Seghiouer et al. [66] declared potentials towards a Hg/HgO reference electrode). The standard electrode potential for this reaction is −0.72 V vs. SHE (−0.925 vs. Ag/AgCl) [67]. Alsabet et al. classified nickel behavior at different potentials in alkaline media as “active” (E < −0.65 V vs. Ag/AgCl), where reversible electro-oxidation of Ni into α-Ni(OH)_2_ occurs, and “passive” (−0.65 V < E < 0.35 V vs. Ag/AgCl), where α-Ni(OH)_2_ irreversibly transforms to β-Ni(OH)_2_. This part of the CV is featureless and is evidence of a passivized surface [68,69,70]. The third region is “transpassive” (E > 0.35 V vs. Ag/AgCl), where oxidation $\mathrm{Ni}{\left(\mathrm{OH}\right)}_{2}+{\mathrm{OH}}^{-}⇌\;\mathrm{NiOOH}+H_{2}O+e^{-}$ occurs. This reaction is represented as the anodic peak at 0.45 V vs. Hg/HgO and a corresponding cathodic peak at 0.42 V vs. Hg/HgO (1 M KOH) in Figure 5 with Ni^+2^ changed to Ni^+3^ oxidation state [66]. After NiOOH was obtained, peaks in the negative potentials disappear, the surface is passivized (no metallic nickel is exposed), and the only electrochemical oxidoreduction that happens during cycling occurs between $\mathrm{Ni}{\left(\mathrm{OH}\right)}_{2}$ and NiOOH [46]. Continuous cycling in alkaline solutions caused growth of the nickel hydroxide layer on the electrode surface [44,45,46,66,71,72,73,74,75,76,77,78]. Alsabet et al. [70] discovered that a surface oxide layer on a Ni electrode is comprised of solid hydroxide Ni(OH)_2_ and solid oxide NiO phases.

Lyons et al. [16] further described the structure of the hydrous nickel oxide thick film obtained by repetitive cycling in alkaline media as “M/MO_x_/MO_a_(OH)_b_(OH_2_)_c_/aquaeous phase”, with M being the metallic phase, MO_x_ being the anhydrous compact oxide layer, and MO_a_(OH)_b_(OH_2_)_c_ being the outer hydrous oxide layer (oxyhydroxide). The peaks’ positions and shapes were heavily dependent on electrode pretreatment and treatment [16,66,70,78]. A rise in the number of cycles causes an increase in full width at half maximum (FWHM) of the redox peaks [16]. The double structure of the peaks (noticed also in Figure 1 and Figure S1) is indicative of the generation of β and γ-NiOOH [66]. Several articles have analyzed the nature of the abovementioned reaction of nickel hydroxides and oxyhydroxides that occur during the cycling of nickel in alkaline media [16,66,70,71,72,73,74,75,76,77,78]. The redox couple ${\mathrm{Ni}(\mathrm{OH})}_{2}$/NiOOH is widely used as a positive electrode in nickel rechargeable batteries [4,5,6,7,8,49,76]. NiO and Ni(OH)_2_ are reported as promising active materials for supercapacitors on a non-nickel substrate [79,80,81,82,83] and on Ni foam or grid as a substrate [84,85,86,87,88]. Metallic nickel interacts with alkaline media in a complex way, including the oxidation and reduction of metallic nickel, nickel oxides, nickel hydroxides, and others [78,89]. All of this information confirms that peaks in the CVs of NiMn_2_O_4_/NF and Ni-Mg ferrites/NF (Figure 1, Figure 2, and Figure S1) at least partially originate from NiO, Ni(OH)_2_, and NiOOH. This is indicated by a constant peak intensity increase and the double structure of the peaks, shapes, and positions of the peaks.

**Figure 6:**
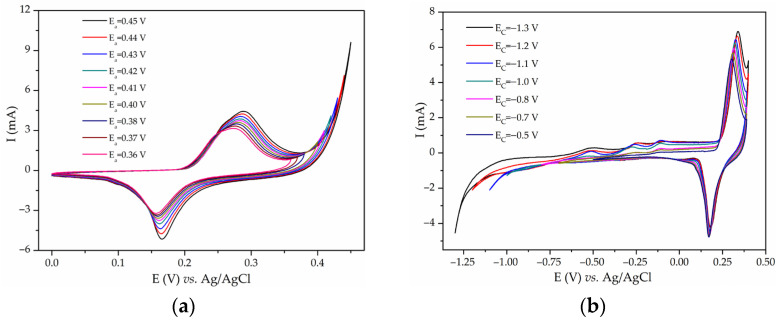
CVs of Ni foam in 3 M KOH at 20 mV/s with (**a**) fixed cathodic limit and (**b**) fixed anodic limit.

The possibility of Ni foam contributing to capacitance values by its own redox reactions is mostly disregarded in the papers reporting on supercapacitor or pseudocapacitive materials. Some papers do actually consider the influence of Ni foam by putting it through one voltammetric cycle, with an almost negligible current response. However, since the Ni(OH)_2_ layer grows during cycling [16,70], those researchers might have made the mistake of not putting uncoated Ni foam through more cycles. The authors of this article underline the problem of galvanostatic charge–discharge (GCD) cycling. If, for example, charge–discharge cycles are obtained with a nickel electrode freshly coated with the active material, there might not be a large portion of the current originating from Ni foam oxidation. But, if the material along with Ni foam is put through tens and hundreds of cycles, in the potential region where nickel electrooxidation occurs, Ni(OH)_2_ formed on the Ni foam surface would probably grow and participate in the sum current with its redox reaction. If the active materials’ peaks are expected in this exact region of potentials where nickel electrooxidation occurs, then Ni foam should be used with caution. The term “synergy” that is sometimes used in the literature, pertaining to the combined capacitance effect of the active material and Ni foam, is controversial since it is hard to distinguish capacitance originating from the active material and from the Ni foam. Active materials should be investigated on planar inert electrodes and then compared to the activity when loaded on Ni foam [48,61,62]. If not, then the results may be overestimated or even completely untrue. Our results concur with the results obtained by Ali et al. [90], who claim that testing on Ni foam gives misleading results and interferes with the active materials’ electrochemistry. The potential window width on Ni foam electrochemistry was investigated. Figure 6a shows the CV of bare Ni foam, cycled in 3 M KOH, with the cathodic limit being fixed at 0.0 V and with the anodic limit values ranging from 0.36 V to 0.45 V. Widening the potential limit into positive potentials increases the peak of Ni(OH)_2_/NiOOH since the oxygen synthesized during OERs reacts with Ni(OH)_2_, enhancing the generation of NiOOH [16]. Figure 6b shows repetitive cycling with the same setup but with an anodic potential limit fixed at 0.4 V and a cathodic potential limit varying in the range −0.5–−1.3 V. With the cathodic potential limit increase in the negative potential region, the HER is more pronounced. Hydrogen reduces NiO and Ni(OH)_2_ to metallic nickel, which reacts with hydroxyl anions in the electrolyte when the potential reverses and forms more Ni(OH)_2_, which, again, generates more NiOOH in the positive potential region.

### 3.2. Nickel Foam “Cleaning”—Experimental Results and Literature Consideration

Some articles proscribe the cleaning of Ni foam by one or a combination of the following processes: degreasing in hot acetone under reflux, etching under the ultrasonic bath in 0.1, 1, 3, or 6 M HCl, ultrasonic treatment of Ni foam in ethanol and deionized water, and rinsing with deionized water [19,24,27,28,29,31,39] to dissolve surface oxides. On the other hand, Bakar et al. [91] discovered that cleaning Ni foam with HCl_aq._ solution leads to the formation of an oxide layer on the Ni foam surface, contrary to the literature that claims that HCl only dissolves surface oxides. They used 0, 1.5, 2.5, 4.0, and 5.0 M HCl solutions for cleaning Ni foam ultrasonically for 10 min. The results showed that upon treatment with HCl, a nickel oxide layer developed on the surface of the nickel electrode, with the thickness of the oxide layer increasing with the concentration of the acid. An increase in surface roughness occurred, which also affects electrochemical energy storage processes. The corrosion of Ni foam was visible after cleaning with higher-concentration HCl [91]. Nickel is amongst the few strong, wrought materials with useful resistance to corrosion by hydrochloric acid, but the rates of corrosion increase with the acid concentration [92,93]. Although Ni can provide some resistance to dilute HCl, corrosion is enhanced by aeration and the presence of oxidizing agents in the acid [94]. On the other hand, when Grden et al. [42] treated Ni foam with a mixture of 30 mL of conc. HNO_3_ and 10 mL of concentrated H_2_SO_4_, the surface of the thus cleaned Ni foam still contained oxides. Yu et al. activated Ni foam with 3 M HCl. They claim that HCl treatment first dissolved the present nickel oxides and then generated a fresh layer of NiO. Activated nickel foam exhibited 10 times greater capacitance [95].

**Figure 7:**
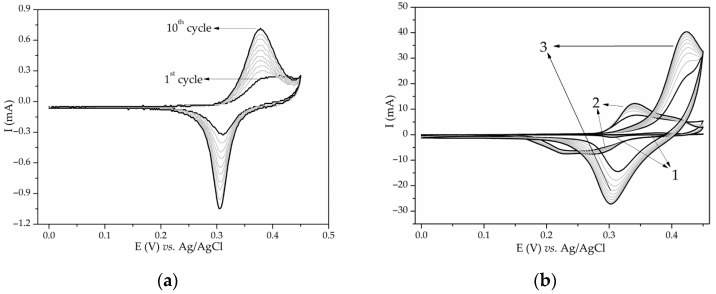
Ten consecutive cycles recorded in 3 M KOH at 20 mV/s of (**a**) untreated Ni foam and (**b**) Ni foam untreated (1) and treated with HCl and ethanol for 10 (2) and 30 min (3).

The effect of dilute HCl on Ni foam was investigated. The scanning rate used in the following measurements was 20 mV/s. Ni foam “as received” exhibited redox peaks in 3 M KOH (Figure 7a,b) that grew with each cycle. After Ni foam was ultrasonically treated with 1 M HCl solution for 10 min, it exhibited increased redox peaks compared to the untreated Ni foam. A change in the shape and FWHM of the peaks with cycling was also evident. Peaks originating from the Ni(OH)_2_/NiOOH redox pair were enhanced multiple times after ultrasonic treatment for 10 or 30 min in 1 M HCl (Figure 7b). The anodic peak current increased from 0.8 mA for “as received” Ni foam to 15 mA for the one treated for 10 min and 40 mA for the one treated for 30 min. A double structured cathodic peak originating from β- and γ-NiOOH [16] is clearly seen in Ni foam treated for 10 min, while these peaks are merged in the CV of Ni foam treated for 30 min. To conclude, in our case, treating Ni foam with 1 M aqueous solution of HCl caused even more enhanced electrooxidation of nickel, probably because of the generation of nickel oxides or because of the increase in surface roughness. We advise careful consideration of the consequences any cleaning process can have on Ni foam before using it as a substrate for electrochemically active material.

### 3.3. Miscellaneous Problems Tied to Ni Foam Use

Batteries and supercapacitors differ in terms of charge storage mechanism, lifetime of the device, specific power, and specific energy. While batteries are superior to supercapacitors in energy density, supercapacitors have greater specific power and are used in electronic equipment when sudden bursts of electrical power are needed [96]. Scientific papers prescribing protocols in the electrochemical investigation of electric energy storage to avoid beginners’ and common mistakes have been published [1,2,97]. Mathis et al. [97] proscribed that cathodic and anodic peaks in CVs with nonlinear GCD curves are an indication of battery-like behavior, where charge storage occurs through Faradaic, diffusion-controlled kinetics, while rectangular CVs and linear GCD curves are an indication of capacitor-like materials, in which case the accumulation of electric charge occurs through surface-controlled kinetics as charge species form an electronic double-layered structure at the electrolyte–material interface. There is a third type, called pseudocapacitive, with electrochemical behavior being neither completely battery-like nor supercapacitor-like. It is a common mistake to assign pseudocapacitive behavior to materials that are clearly, by their electrochemical sign in the CVs and GCD curves, battery-type or supercapacitor-type inside the never-ending citing cycle. This is followed by using an incorrect unit. Namely, the charge storage ability of battery-type materials is expressed through a specific capacity, and its dimensions are I × t/m, while its units are generally mAh/g. Analogous ability in capacitor-like materials is specific capacitance, which is mainly reported in dimensions C/m and units F/g. Therefore, a researcher should analyze materials and report accordingly. Many papers that include the electrochemical behavior of the materials coated on Ni foam have clearly differentiated anodic and cathodic peaks and nonlinear GCD curves, and, nevertheless, they are assigned as pseudocapacitors, and their charge storage ability is expressed in F/g. This article was written in the same manner, with a battery-type material being considered as a capacitive one, only to establish a comparison between ours and other researchers’ work.

Immersion of Ni foam can also have significant effects on the obtained results. Partial immersion causes the electrolyte to climb up the foam because of capillary forces. This imports error because there are more active sites participating in the reaction than estimated [48]. But also, the authors of the current paper remark that if the researcher uses ordinary alligator clips to hold Ni foam, there is a possibility that they will be wet with electrolyte and may corrode. Zheng et al. [48] claim that capillary climb up, along with the immersion effect, varies depending on the nature of the active material. In their conclusion, Zheng et al. [48] described that using foam-type electrodes is “problematic” and provides experimental inconsistencies.

NFs have been widely used as current collecting substrates and support matrices in water splitting reactions. Hydrothermal (HT) is one of the most widely employed techniques used to synthesize nanostructured materials [98]. Most of the published works overlook the effect of nickel corrosion on electrocatalysts directly fabricated on NFs via the HT method [99]. Nickel substrates do corrode, with ions diffusing into the reaction solution and incorporating into the fabricated electrocatalysts, which then affects their electrocatalytic performances [99]. Ni foams are prone to corrosion and chemical etching during hydrothermal synthesis at temperatures above 170 °C. Additives such as urea may hydrolyze during the HT process and raise the pH value, which oxidizes nickel ions obtained by etching into Ni(OH)_2_. Cobalt carbonate hydroxide had 30% Ni/Co after HT at 150 °C [99]. When nickel ions are deposited onto the surface of the active material, the well-known Ni(OH)_2_/NiOOH reaction may contribute to the sum current and give falsely enhanced results to the investigated active material.

Ni foam alone requires a relatively low overpotential of 0.217 V in an HER to produce a current density of 10 mA cm^−2^, which is comparable to some reported electrocatalysts but not as good as Pt/C. Ni foam has relatively poor OER activity, with an overpotential of 337 mV at the current density of 10 mA cm^−2^, which is comparable to or even better than many other reported bifunctional water-splitting catalysts [15]. Bu et al. discovered that sometimes even bare Ni foam showed better electrocatalytic performance for OER catalysis than some investigated active materials coated on Ni foam [99].

Oxidation reactions of nickel may enhance OER activity. The OER electrocatalytic activity of NiCoP was attributed to the formation of Ni–Co oxo/hydroxo species on the NiCoP electrode surface due to the oxidation of Ni and Co atoms [100].

Another problem with using Ni foam as a substrate in water splitting reactions is the electrode surface calculation. The electrode surface area is mostly assessed as a geometric area, which results in exaggerated values. There are other ways of calculating the surface area, with the most suitable one being electrochemically active surface area (ESCA) calculated through the capacitance of the double layer of the electrode (C_DL_) [101]. However, the calculated ECSA constitutes all Faradaic and non-Faradaic active sites, which do not necessarily participate in the observed electrochemical reaction [48], so ECSA can cause exaggerated values for the active surface. Zheng et al. [48] also claim that since ECSA depends on the chemical species of the investigated surface, all metallic Ni should be turned to hydroxide and oxide forms, i.e., the surface should be completely passivized. Even then, the estimation of ECSA is difficult because of the presence of various nickel oxides, hydroxides, and oxyhydroxides on the surface of the Ni foam [48].

The authors would also like to address the problematic mass loading of active materials on Ni foam. Usually, when planar electrodes are used, thick electrode ink is made by dispersing active material, a binder (Nafion solution in alcohol or PVDF solution in pyrrolidone), and a conductivity enhancer (carbon black) in a mass ratio of 85:10:5 in a small quantity of liquid (usually around 100 μL of ethanol, N-methyl-2-pyrrolidone, or some other solvent) [102]. By taking an adequate volume of ink, the desired mass loading on the electrode can be obtained. Nevertheless, such electrode ink was too dense to be applied on Ni foam. Therefore, it had to be diluted five times to adequately cover Ni foam. With such diluted ink, much of the ink dripped down from Ni foam, causing material loss. Because of these difficulties, experimental mass loading was inconsistent, and it varied between 0.5 and 11 mg.

Another practical problem is that a variety of Ni foams used in research practice differ in thickness and purity, which can affect catalyst performance. Impurities such as iron may influence electrocatalytic or energy storage performance, and a difference in thickness or pore size heavily influences the surface of the Ni foam substrate [48,101]. Also, purity affects the efficiency of surface activation techniques [103].

## 4. Conclusions

Using Ni foam as a substrate in the investigation of supercapacitor materials comes with a lot of experimental problems. The electrochemical oxidoreduction of nickel in alkaline media impairs the use of Ni foam as a substrate if the potential window where active materials exhibit energy storage is the same as where NiO or Ni(OH)_2_ oxidoreduction occurs. The surface of nickel electrodes is almost never without oxides, which are electroactive, and the oxide layer grows with cycling and affects the results more with each cycle. The consequences of Ni foam “cleaning” with hydrochloric acid are debatable. Treating it with high-concentration acid solutions causes nickel oxide formation, or if the acid is dilute, it does not completely clean the nickel surface of its oxides. Other problems such as Ni foam immersion and purity need to be carefully considered. The electrode surface area calculation is troublesome, and there is evidence that Ni foam, when used in hydrothermal synthesis, can contaminate the active material. The authors suggest that researchers should reflect on the abovementioned problems when using Ni foam as a substrate in electrochemical investigations. Ni foam use is justified when (1) the monitored potential window is not where Ni electrooxidation occurs, (2) when NiO and Ni(OH)_2_ derived from Ni foam are the intended electroactive materials, and (3) when Ni foam is used in tandem with the active materials but the possible electrooxidation of nickel isconsidered. All measurements should be performed both with and without the active materials on Ni foam using consistent methods to prevent the overestimation of capacitance. Another helpful procedure would be to test active materials both on Ni foam and planar electrodes and compare the obtained results.

## Data Availability

The data presented in this study are available on request from the corresponding author. The data are not publicly available due to ongoing research.

## Supplementary Materials

The following supporting information can be downloaded at: https://www.mdpi.com/article/10.3390/ma17061292/s1, Figure S1: CVs of Mg_x_Ni_1-x_Fe_2_O_4_ with x being 0.1, 0.3, 0.5, 0.7, 0.9, and 1.0 a-f, respectively, in 3 M KOH.
