# Supplementary material for: Supercapacitor Electrodes: Is Nickel Foam the Right Substrate for Active Materials?
Source: Materials (Basel). 2024 Mar 11;17(6):1292. doi: 10.3390/ma17061292 (PMC10971949; doi:10.3390/ma17061292)
Supplement: Supplementary file 1 [file materials-17-01292-s001.zip › materials-2890203-supplementary.pdf]

Supplementary

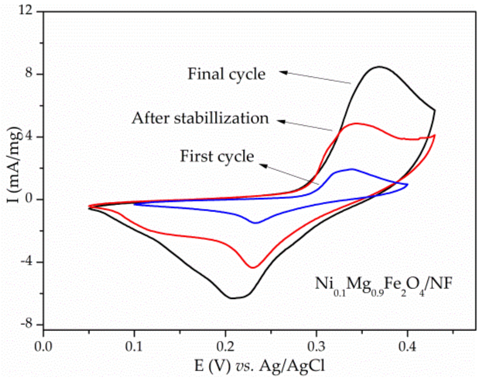

(a)

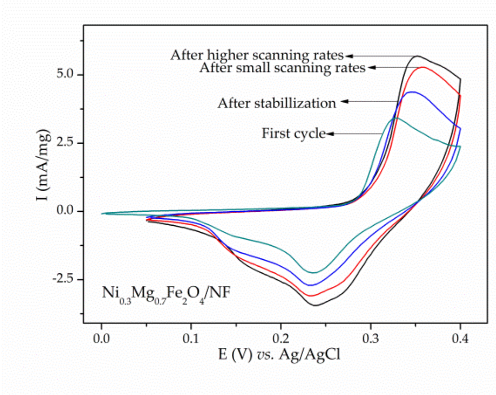

(b)

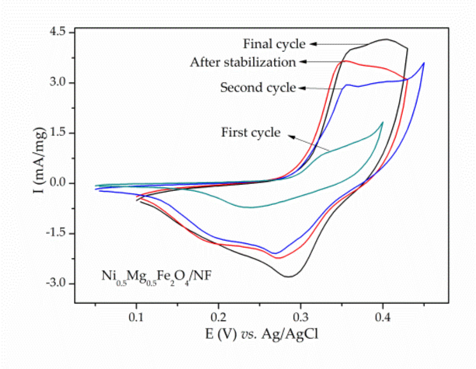

(c)

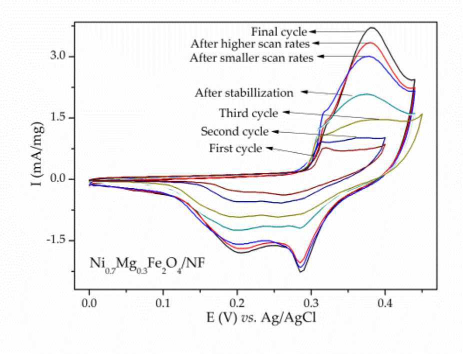

(d)

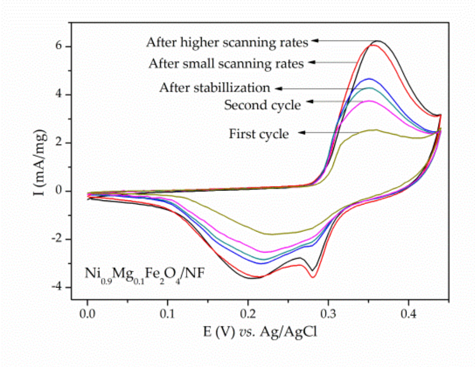

(e)

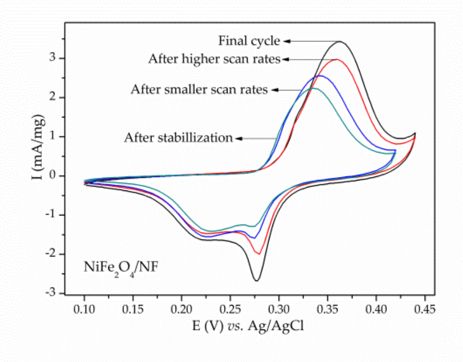

(f)

Figure S1 CVs of $Mg_xNi_{1-x}Fe_2O_4$ with x being 0.1, 0.3, 0.5, 0.7, 0.9, and 1.0 a-f, respectively, in 3 M KOH
